# Supplementary material for: MetaRibo-Seq measures translation in microbiomes
Source: Nat Commun. 2020 Jun 29;11:3268. doi: 10.1038/s41467-020-17081-z (PMC7324362; doi:10.1038/s41467-020-17081-z)
Supplement: Supplementary file 10 — Supplementary Data 7 [file 41467_2020_17081_MOESM10_ESM.zip › File2/Confidence_High_Taxonomy/334622_out.krona.html]

Javascript must be enabled to view this page.

members
magnitude
magnitudeUnassigned
count
unassigned
taxon
rank

334622\_out

22

2
22
superkingdom

74201
1
phylum

class
1
203494

order
1
48461

family
1
1647988

239934
1
genus


SRS146813\_contig\_number\_4236
species
1
239935

phylum
21
976

21
200643
class

order
21
171549

815
21
family


SRS024140\_contig\_number\_contig-100\_919.919
genus
21
816
1


SRS016349\_contig\_number\_contig-100\_25236.25236SRS018351\_contig\_number\_contig-100\_2227.81886SRS019685\_contig\_number\_43133SRS020622\_contig\_number\_6672SRS049900\_contig\_number\_10287SRS050801\_contig\_number\_contig-100\_1544.29591SRS064232\_contig\_number\_contig-100\_70.38262SRS064757\_contig\_number\_21039SRS104036\_contig\_number\_6317SRS104485\_contig\_number\_199SRS1055056\_contig\_number\_11921SRS148159\_contig\_number\_54489SRS893300\_contig\_number\_12965
821
13
species


SRS020869\_contig\_number\_32246
species
1
2292001


SRS075078\_contig\_number\_32144
1
species
818


SRS015794\_contig\_number\_13435
1
species
1263042


SRS075878\_contig\_number\_17663
310297
1
species

2
357276
species

SRS014412\_contig\_number\_15144SRS1041032\_contig\_number\_contig-100\_2406.90615

2292943
1
species

SRS063040\_contig\_number\_36725
